# Strain-Specific Antagonism of the Human H1N1 Influenza A Virus against Equine Tetherin

**DOI:** 10.3390/v10050264

**Published:** 2018-05-16

**Authors:** Meiyue Wang, Zhenyu Zhang, Xiaojun Wang

**Affiliations:** State Key Laboratory of Veterinary Biotechnology, Harbin Veterinary Research Institute, Chinese Academy of Agricultural Sciences, Harbin 150069, China; wangmeiyue@caas.cn (M.W.); zhangzhenyu@caas.cn (Z.Z.)

**Keywords:** influenza A virus, HA, NA, tetherin, equine, interspecies restriction

## Abstract

Tetherin/BST-2/CD317 is an interferon-induced host restriction factor that can block the budding of enveloped viruses by tethering them to the cell surface. Many viruses use certain proteins to counteract restriction by tetherin from their natural hosts, but not from other species. The influenza A virus (FLUAV) has a wide range of subtypes with different host tropisms. Human tetherin (huTHN) has been reported to restrict only specific FLUAV strains and the viral hemagglutinin (HA) and neuraminidase (NA) genes determine the sensitivity to huTHN. Whether tetherins from other hosts can block human FLUAV is still unknown. Here, we evaluate the impact of equine tetherin (eqTHN) and huTHN on the replication of A/Sichuan/1/2009 (H1N1) and A/equine/Xinjiang/1/2007 (H3N8) strains. Our results show that eqTHN had higher restriction activity towards both viruses, and its shorter cytoplasmic tail contributed to that activity. We further demonstrated that HA and NA of A/Hamburg/4/2009 (H1N1) could counteract eqTHN. Notably, our results indicate that four amino acids, 13T and 49L of HA and 32T and 80V of NA, were involved in blocking the restriction activity of eqTHN. These findings reveal interspecies restriction by eqTHN towards FLUAV, and the role of the HA and NA proteins in overcoming this restriction.

## 1. Introduction

Viruses and their hosts undergo coevolution and adaptation over long time scales and most viruses have a defined range of hosts. For example, most retroviruses have strict host tropism and can rarely jump from one species to another. However, the influenza A virus (FLUAV) of the *Orthomyxoviridae* can infect different hosts and the virus can transmit between species [1,2]. To successfully replicate in host cells, viruses need to counteract various host restriction factors at different replication steps. It is evident from several reports that host restriction factors, such as apolipoprotein B mRNA-editing enzyme catalytic subunit 3 proteins (APOBEC3) [3,4,5], tripartite motif protein 5a (TRIM5a) [6], SAM domain and HD domain-containing protein 1 (SAMHD1) [7,8], and tetherin [9] play important roles in blocking interspecies transmission of retroviruses. As with several other restriction factors, like interferon-induced transmembrane proteins (IFITMs), tetherin has been shown to have broad antiviral activity against different enveloped viruses from various virus families including human immunodeficiency virus 1 (HIV-1), Ebola virus and human herpes virus 8 (HHV8) [10,11,12]. 

Tetherin is a type II single-pass transmembrane protein with a cytoplasmic tail, a transmembrane domain, an extracellular domain, and a putative glycophosphatidylinositol (GPI) lipid anchor from its N terminus to C terminus [13,14,15]. Tetherin mainly blocks enveloped viruses through a shared mechanism tethering them to the cell membrane [16], while different viruses take different measures to antagonize its restriction [17]. Human tetherin (huTHN) was first reported as being able to inhibit egress of HIV-1 viral particles deficient in the viral membrane protein Vpu [9]. Vpu can downregulate huTHN from the cell surface by targeting it for proteasomal or lysosomal degradation [18,19]. Other enveloped viruses including HHV8, Ebola virus, simian immunodeficiency virus (SIV) and equine infectious anemia virus (EIAV) are also found to be restricted by tetherin and these viruses in different hosts have different counteraction mechanisms. For instance, SIV uses its nef to counteract simian tetherin, while EIAV env plays this role in overcoming equine tetherin (eqTHN) [20,21,22]. 

Interspecies transmission of animal FLUAV to humans may have the potential to cause pandemics and can result in severe disease and huge economic loss, such as the pandemics that occurred in 1918 and 2009. FLUAV is an enveloped virus with a segmented negative strand RNA genome. Two viral proteins play a significant role in interspecies transmission of FLUAV:HA, which is responsible for recognizing and binding with the sialic acid (SA) receptor on the surface of host cells; and NA, which helps the release of virions [2,23]. Mutations in HA can alter its preference from the α2,3 to the α2,6 SA receptor in order to adapt to humans [24]. Furthermore, compensatory mutations in NA may also be selected in order to achieve an optimal balance for effective viral transmission [25]. The role of tetherin in the inhibition of FLUAV budding has been investigated and some studies show that tetherin has no function in this area [26,27]. While in a recent study it was confirmed that the sensitivity of FLUAV to huTHN is strain specific, HA and NA are known to confer tetherin resistance to certain pandemic viruses [28]. To date, there are no studies on the activity of tetherins from different species that block FLUAV. Tetherin has been shown in many cases to have species-specific antiviral activity, especially in the restriction of retroviruses. An example is huTHN, which has broad anti-retrovirus activity, but can only be neutralized by HIV-1 Vpu protein [9,18,19]. Similarly, the anti-retrovirus activity of eqTHNs can only be counteracted by EIAV envelope protein but not other viruses [20]. It is interesting that for FLUAV, the activity of huTHN is limited to certain isolates from humans, but not isolates from other animals. It would be of great value to know, on one hand, whether tetherin from other animals (such as eqTHN) has anti-FLUAV activity and whether it is species specific; and on the other hand, by which mechanism FLUAV is able to counteract the anti-retroviral activity of tetherin.

In the present study, we find that eqTHN, but not huTHN, has restriction activity towards human FLUAV A/Sichuan/1/2009 (H1N1) and equine FLUAV A/equine/Xinjiang/1/2007 (H3N8). The relatively shorter cytoplasmic tail domain of eqTHN determines its molecular activity. HA and NA of A/Hamburg/4/2009 (H1N1) can counteract both huTHN and eqTHN. Importantly, our results demonstrate that 13T and 49L in HA, and 32T and 80V in NA can help the virus A/Sichuan/1/2009 (H1N1) to counteract eqTHN. By contrast, these sites have no impact on antagonism against huTHN.

## 2. Materials and Methods

### 2.1. Cell Culture 

Human embryonic kidney (HEK) 293T cells and Madin-Darby canine kidney (MDCK) cell lines were maintained in Dulbecco’s high-glucose modified Eagle medium (HyClone, Logan, Utah, USA) supplemented with 10% fetal calf serum (Sigma, St. Louis, MO, USA) and antibiotics (100 units/mL penicillin and 100 μg/mL streptomycin; Thermo Fisher, Waltham, MA, USA). Cell cultures were incubated at 37 °C in an atmosphere with 5% CO_2_.

### 2.2. Plasmids 

pLPCX, the MLV Gag polymerase expression vector pCGP, and pVSV-G were purchased from Clontech (Felicia, CA, USA). Plasmids expressing huTHN and eqTHN proteins with an in-frame N-terminal HA were generated by PCR. PCR-generated fragments were then cloned into the pLPCX vector via the NotI and XhoI restriction sites. To establish MDCK cell lines expressing tetherin mutants, including an eqTHN GPI deletion mutant with a C-terminal 26 amino acid deletion (pLPCX_eqTHN_del_GPI), an huTHN mutant with an N-terminal 14 amino acid deletion (pLPCX_huTHN_del-14), and a chimera pLPCX_hu_eqTHN, in which the N-terminal 14 amino acids of huTHN were added to the N terminus of eqTHN, all mutant plasmids were constructed according to the online In-Fusion ^®^HD Cloning Kit User Manual (http://www.clontech.com/CN/Products/Cloning_and_Competent_Cells/Cloning_Kits/xxclt_searchResults.jsp). Briefly, the fragments of the pLPCX vector and each target gene were amplified with a 15 bp homologous arm and were then fused using the In-Fusion HD Enzyme (Clontech, Felicia, CA, USA). To create the pLPCX_hu_eqTHN plasmid, pLPCX_huTHN was used as the template to amplify the pLPCX vector, including an HA tag and the N-terminal 14 amino acids of huTHN. This sequence was then fused with the eqTHN fragment. To obtain the pLPCX_eqTHN_del_GPI and pLPCX_huTHN_del_14 plasmids, eqTHN_del_GPI and huTHN_del_14 sequences were amplified using pLPCX_eqTHN and pLPCX_huTHN as the templates, respectively, and were fused with the pLPCX-HA vector. The 8 plasmids for rescuing the virus A/Sichuan/1/2009 (H1N1) were kindly provided by Dr. Hualan Chen, Harbin Veterinary Research Institute, Chinese Academy of Agricultural Sciences. The 8 plasmids for rescuing the virus A/equine/Xinjiang/1/2007 (H3N8) were constructed in-house and stored at the laboratory. The plasmids HA_I49L, HA_A13T, NA_I32T and NA_M80V, for generating mutant viruses, were produced by PCR using mutation primers. All primer sequences are available upon request. All constructed plasmids were confirmed by sequencing. 

### 2.3. Creation of Stable Cell Lines

To create MDCK cell lines stably expressing the proteins HA, HA-eqTHN, HA-huTHN, HA-eqTHN_del_GPI, HA-huTHN_del_14, or HA-hu_eqTHN, 9 μg target plasmid together with a total of 12 μg helper plasmids (9 μg pCGP and 3 μg pVSV-G), were co-transfected into 293T cells cultured in a 10-cm dish by the calcium phosphate transfection method. 48 h post transfection, the supernatant was collected and centrifuged at 1000× *g* for 5 min to remove cell debris leaving the viruses. These harvested viruses were used to infect MDCK cells. The cell lines were then selected using 5 μg/mL puromycin and purified by limited dilution.

### 2.4. Western Blotting 

For assessing protein expression, cell lines were lysed using a RIPA Lysis Buffer (Solarbio, Beijing, China), and centrifuged at 13,000× *g* and 4 °C for 10 min. Samples were taken from the supernatant, separated on 4–12% gels by SDS-polyacrylamide gel electrophoresis (SDS-PAGE) and transferred onto nitrocellulose membranes. Membranes were blocked with 5% milk powder in Tris-buffered saline (TBS) for 2 h. Incubation with the first anti-mouse HA (Sigma, 1:10,000) and antibody-TBST (TBS plus 0.05% Tween20) was performed for 2 h at room temperature (RT), followed by washing three times with TBST. The secondary antibody (Sigma, 1:10,000) was then applied and samples were incubated at RT for 1 h. Subsequently, membranes were washed three times for 10 min with TBST. Signals were detected by a LI-COR Odyssey Imaging System (LI-COR, Lincoln, NE, USA). 

### 2.5. Production of Viruses and Recombinant Viruses

The viruses, including A/Sichuan/1/2009 (H1N1), A/equine/Xinjiang/1/2007 (H3N8), and recombinant viruses were generated through the reverse genetic method as previously described [29,30]. Viruses were proliferated using 9-day-old embryonated hen eggs, which were incubated for 72 h at 35 °C. The SC09 (A/Sichuan/1/2009)-based 6 + 2 reassortant expressing the HA and NA of A/Hamburg/4/2009 (H1N1), the SC09-based 7 + 1 reassortant viruses expressing the single HA or NA of A/Hamburg/4/2009 (H1N1), and mutants with single point mutations in HA or NA were generated by using mutant plasmids and propagated as above. All vRNA was extracted from the viruses using the QIAamp viral RNA mini kit (Qiagen, Dusseldorf, Germany). cDNA was then synthesized from vRNA by reverse transcription-PCR with gene-specific primers. The identity of these viruses was confirmed by sequencing.

### 2.6. Infection of Tetherin-Expressing Cells with FLUAV

For infection experiments, tetherin-expressing MDCK cells were seeded in 6-well plates. When the density of cells reached about 70%, the culture medium was removed and the cells were gently washed twice with preheated phosphate-buffered saline (PBS) and then incubated with DMEM supplemented with 1 μg/mL TPCK (Sigma, St. Louis, MO, USA), 0.25% BSA (Roche, Basel, Switzerland) and FLUAV at different multiplicities of infection (determined by plaque-forming units assay). After 1 h of incubation at 37 °C, the infection medium was removed, the cells were gently washed with preheated PBS, and DMEM supplemented with 1 μg/mL TPCK and 0.25% BSA was added. Finally, the supernatants were collected at 36, 40, 44, 48 and 52 h post-infection and debris was removed by centrifugation at 1000× *g* for 5 min. The amount of virus present in each supernatant was determined by an in-house ELISA method.

### 2.7. Quantitative ELISA for Determination of Virus Production

A 96-well microtiter plate (Costar, Bodenheim, Germany) was coated with 1 μg/well of mouse monoclonal anti-FLUAV nucleoprotein (NP) antibody (gifted by Dr. Ning Fu from Southern Medical University, Guangzhou, China) in PBS, sealed and incubated overnight at 4 °C. The plate was washed three times with washing buffer (PBS containing 0.1% Tween-20, PBST) and blocked with 200 μL 5% calf serum at 37 °C for 2 h. After washing three times with PBST buffer, 100 μL each of a series of diluted virus samples in dilution buffer (PBS containing 10% calf serum and 0.1% Triton X-100) was added to each well, and the plate was incubated at 37 °C for 1 h. Following a further washing step, 100 μL of a 1:2000 dilution of horseradish peroxidase (HRP)-conjugated anti-FLUAV NP protein mAb was added to each well. After incubation at 37 °C for 30 min, the plate was washed again and incubated with freshly prepared tetramethylbenzidine (TMB) peroxidase substrate (Galaxy Bio, Princeton, New Jersey, USA) for 10 min at RT. The reaction was stopped by adding 50 μL of 2 M H_2_SO_4_ and the optical density at 450 nm (OD450) was measured using the VersaMax Microplate Reader (BioTek, Winooski, VT, USA). Dilution buffer was used as blank control and the purified 1:2 diluted FLUAV NP protein was used as a standard. The virus production was calculated according to the standard curve. 

### 2.8. Focus Formation Units Assay

Viral titers were determined on MDCK cells by using the focus formation units (FFU) assay. In brief, MDCK cells (3 × 10^4^ cells/well) were seeded in 96-well plates and incubated for 12–14 h at 37 °C in 5% CO_2_. Serial 10-fold dilutions of viral supernatant in DMEM containing 2.5 μg/mL Trypsin, 0.25% BSA, 1% penicillin/streptomycin were added to MDCK cells (50 μL/well). After 1 h of incubation, the medium were then replaced with 100 μL of 1% Avicel overlay (Solarbio, Beijing, China) in DMEM containing 2.5 μg/mL Trypsin, 0.25% BSA and 1% penicillin/streptomycin. After 24 h of incubation, the cells were subsequently washed twice gently with PBS and fixed with 4% paraformaldehyde (100 μL/well, Beyotime Biotechnology, Shanghai, China) for 15 min at RT. The cells were then washed twice gently and incubated with 0.1% Triton-X100 (100 μL/well) for 15 min at RT. After blocking with blocking buffer (BB) (50 μL/well; 0.5% Tween, 3% BSA in PBS) for 2 h at RT, the cells were incubated with the primary antibody (anti-FLUAV NP mouse antibody) and the secondary antibody (anti-mouse-HRP from KPL, 50 μL/well) for 1 h at RT. With a further wash, the cells were incubated with 50 μL of True Blue substrate (KPL, Milford, MA, USA) for about 10 min until blue spots from infected cells appeared. Foci were counted and viral titers were calculated as FFU per mL. 

### 2.9. Structure Prediction 

The structures of HA from A/Sichuan/1/2009 (H1N1) and A/Hamburg/4/2009 (H1N1) were predicted using the online Phyre2 Protein Fold Recognition Server (http://www.sbg.bio.ic.ac.uk/phyre2/html/page.cgi?id=index), which predicted them with >90% confidence. Structures were subsequently analyzed using Swiss-PBD Viewer 4.10 software (Swiss Institute of Bioinformatics, Basel, Switzerland).

### 2.10. Confocal Laser Scan Microscopy

The cell lines were plated into the cell culture capsule (NEST, Jiangsu, China). When the density of the cells reached about 80%, the cells were washed with PBS three times and were then fixed with 1 mL 4% paraformaldehyde followed by incubation for 30 min at RT. The cells were then washed a further three times and 1 mL 0.1%Triton-X100 was added to each sample and the samples were incubated at RT for 20 min. After blocking with 5% milk powder–phosphate-buffer for 2 h, the cells were incubated with an anti-rabbit HA antibody (Sigma, 1:200) for 1 h at RT, then for a further hour with anti-rabbit secondary antibody conjugated with fluorescein isothiocyanate (FITC) (Sigma, 1:200). Finally, the cells were dyed with anti-fade-46-diamidino-2-phenylindole (DAPI, Beyotime Biotechnology, Shanghai, China) and observed using a Leica DM-IRE2 confocal microscope (Wetzlar, Germany). For subcellular location of tetherins after viral infection, the cell lines were seeded into the capsule, and when the density of the cells reached 70–80%, they were infected with SC09 or SC09_rHBG HA/NA_ at a multiplicity of infection (MOI) of 0.1. The supernatant was removed 48 h after infection, and cells were then treated as above. 

### 2.11. Statistical Analysis 

Results were analyzed using unpaired *t* tests for comparing two groups and using an ANOVA followed by a post-hoc test for comparing three groups in the GraphPad Prism 5.01 software (University of California San Diego, San Diego, California, USA) (***, *p* < 0.001; **, *p* < 0.01; *, *p* < 0.05; ns, *p* > 0.05).

## 3. Results

### 3.1. EqTHN Was Able to Block the Release of both Human FLUAV SC09 and Equine FLUAV XJ07

**Figure 1:**
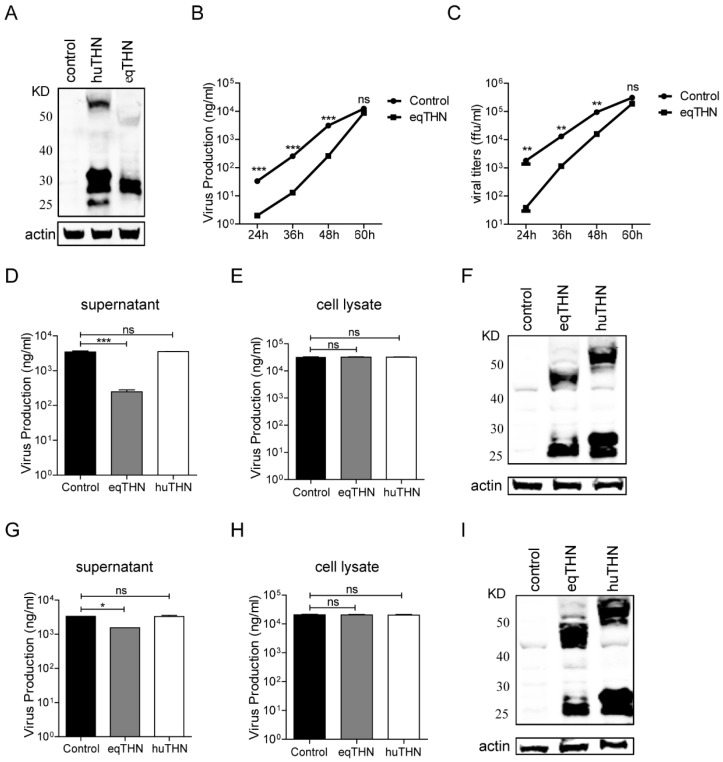
Restriction activity of human tetherin (huTHN) and equine tetherin (eqTHN) towards human H1N1 SC09 and equine H3N8 XJ07. (**A**) Stable expression of HA-tagged huTHN and eqTHN in Madin-Darby canine kidney (MDCK) cells. Cells were lysed in radio immunoprecipitation assay (RIPA) lysis buffer and proteins were separated by 4–12% SDS-PAGE, followed by immunoblotting with anti-HA antibody to detect the HA-tagged tetherins; (**B**) The amount of SC09 virus released from MDCK and eqTHN-expressing MDCK cells at 24 h, 36 h, 48 h and 60 h post infection with SC09 at a multiplicity of infection (MOI) of 0.1, as determined using ELISA. At 1 h post infection, the excess input viruses were removed by washing using preheated phosphate-buffered saline (PBS), and at 24 h, 36 h, 48 h and 60 h post-infection, the supernatants were harvested and the amount of virus released was determined by ELISA. The results of a single experiment performed with triplicate samples are shown and results were subsequently confirmed in three separate experiments. Error bars indicate standard deviations (SD); (**C**) The amount of SC09 virus released from MDCK and eqTHN-expressing MDCK cells at 24 h, 36 h, 48 h and 60 h post-infection with SC09 at an MOI of 0.1, as determined by the focus formation units assay. At 1 h post infection, input viruses were removed by washing with PBS, and at 24 h, 36 h, 48 h and 60 h post-infection, the supernatants were harvested and titers determined using the focus formation units assay. The results of a single experiment performed with triplicate samples are shown and results were subsequently confirmed in three separate experiments. Error bars indicate standard deviations (SD); (**D**–**F**) The amount of SC09 virus released from MDCK, huTHN and eqTHN-expressing MDCK cells at 48 h post infection with SC09 at an MOI of 0.1. At 1 h post-infection, the excess input viruses were removed by washing using preheated PBS, and at 48 h post-infection, the supernatants were harvested and the amount of virus released was determined by ELISA. At the same time, cells were lysed and nucleoprotein (NP) was detected by ELISA and tetherin expression determined by western blot. The results of a single experiment performed with triplicate samples are shown, results were later confirmed in three separate experiments. Error bars indicate standard deviations (SD); (**G**–**I**) The amount of XJ07 virus released from MDCK, huTHN and eqTHN-expressing MDCK cells at 48 h post infection with XJ07 at an MOI of 0.2. Infection and detection were carried out as described for panels D–F. The results of a single experiment performed with triplicate samples are shown and results were subsequently confirmed in three separate experiments. Error bars indicate standard deviations (SD).

FLUAV has been the subject of inconsistent data regarding its sensitivity to tetherin. Therefore, we first addressed whether huTHN and eqTHN proteins exerted an influence on FLUAV release. For this purpose, three MDCK cell lines constitutively expressing either an HA tag, an N-terminally HA-tagged huTHN or eqTHN protein, were generated. High levels of expression of huTHN and eqTHN proteins in cell lines were detected by western blotting (Figure 1A). HuTHN and eqTHN proteins were potentially glycosylated, leading to them running as variably sized smears. The cell line expressing the HA tag was used as a negative control to remove HA tag interference during viral budding. 

The eqTHN-expressing and control MDCK cells were then each infected with FLUAV A/Sichuan/1/2009 (H1N1) (abbreviated SC09) at an MOI of 0.1. The amount of virus released was determined at 24 h, 36 h, 48 h and 60 h post-infection using a quantitative ELISA as well as a focus formation units assay, which is widely used to measure the viral titer of infectious particles. Both the ELISA and FFU assays showed that eqTHN could significantly inhibit the replication of SC09 before 60 h post-infection (Figure 1B,C) and correlated well with each other, indicating that the quantitative ELISA could be used as an indirect measure of viral titer. 

In order to compare inhibitory activity of huTHN and eqTHN towards FLUAV and the inhibitory mechanism, the cells that could stably express huTHN and eqTHN MDCK cells and the control cells were each infected with SC09 at an MOI of 0.1. The amount of viruses released and viral NP protein in the cells was determined at 48 h post infection using the quantitative ELISA.

Our results showed that the SC09 output present in the supernatant in the eqTHN-expressing MDCK cell line was about 20 fold lower than in the control cell line at 48 h post-infection. HuTHN, however, could not inhibit the production of SC09 (Figure 1D). The expression levels of endocellular NP in these three cell lines were almost the same (Figure 1E) and tetherin also expressed well (Figure 1F), which meant that neither eqTHN nor huTHN affected the production of viral protein and tetherin functioned by restricting the release of FLUAV. To extend these observations, in a further experiment, the virus A/equine/Xinjiang/1/2007 (H3N8) (abbreviated XJ07) was introduced to the same three cell lines at an MOI of 0.2. Quantification of the number of XJ07 viruses released into the supernatant showed no differences between huTHN-expressing and control cell lines. A roughly 3-fold reduction of XJ07 release that did not affect the expression of viral NP or cellular tetherins was observed in eqTHN-expressing MDCK cell lines compared with the control, which was statistically significant when assessed using an ANOVA, followed by a post-hoc test. Quantification of the number of XJ07 viruses released into the supernatant showed no differences between huTHN-expressing and control cell lines (Figure 1G–I). Because of the importance of polymerase activity on FLUAV replication, we performed a polymerase assay to test whether tetherin could affect polymerase activity and caused the observed differences in restriction activity in eqTHN and huTHN towards both SC09 and XJ07. The expression of eqTHN or huTHN by transient transfection with increasing dose in HEK293T cells, which lack endogenous tetherin, had no impact on SC09 and XJ07 polymerase activity [31]. Taken together, these results suggested that eqTHN had higher activity than huTHN on restricting the release of SC09 and XJ07. 

### 3.2. The Shorter Cytoplasmic Tail Domain of EqTHN to Some Extent Facilitated Its Stronger Restriction Activity

**Figure 2:**
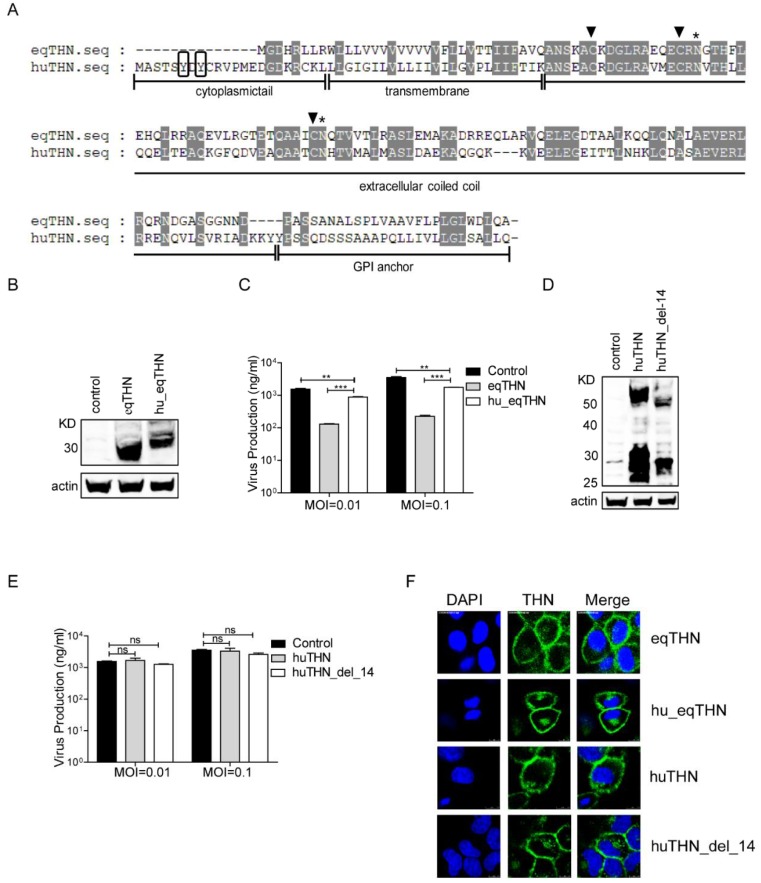
The shorter cytoplasmic tail domain of eqTHN contributed to its stronger restriction activity. (**A**) Amino acid sequence alignment of huTHN and eqTHN. The domains displayed include a cytoplasmic tail, a transmembrane region (TM), an extracellular coiled-coil domain, and a putative glycophosphatidylinositol (GPI) anchor. Identical amino acids are shaded in light gray. The dual tyrosine motif (Y6–Y8) at the cytoplasmic tail domain of huTHN is boxed in black. Three Cys residues in the extracellular domain are marked with black triangles. Two putative N-glycosylation sites in the extracellular domain are marked with asterisks; (**B**) Stable expression of HA-tagged hu_eqTHN in MDCK cells. Cells were lysed and the sample was treated as described for panel A in Figure 1; (**C**) The amount of SC09 released from MDCK, eqTHN-expressing and hu_eqTHN-expressing MDCK cells at 48 h post-infection at MOIs of 0.01 and 0.1. Infection and detection were carried out as described as above. The results of a single experiment performed with duplicate samples are shown, and results were subsequently confirmed in three separate experiments. Error bars indicate standard deviations (SD); (**D**) Stable expression of HA-tagged huTHN_del_14 in MDCK cells. Cells were lysed and the sample was treated as described for panel A in Figure 1; (**E**) The amount of SC09 released from MDCK, huTHN-expressing and huTHN_del_14-expressing MDCK cells at 48 h post infection with SC09 at MOIs of 0.01 and 0.1. Infection and detection were carried out as described as above. The results of a single experiment performed with duplicate samples are shown, and results were subsequently confirmed in three separate experiments. Error bars indicate standard deviations (SD); (**F**) Subcellular localization of tetherin and mutants on the cell surface with scale bars indicating 10 μm. HA-tagged huTHN, eqTHN, huTHN_del_14 and hu_eqTHN-expressing MDCK cells were stained with an anti-HA antibody (green) and nuclei were stained with DAPI (blue). The cells were subsequently analyzed using confocal microscopy.

The structure of a protein is of critical importance to any antiviral function it may have. By analyzing the sequence features of both eqTHN and huTHN through amino acid sequence alignment, we found that there was only about 40% sequence similarity between them and equivalent sites were in different places in the two proteins (Figure 2A). In both species, three cysteine residues, which are important for dimer formation, and two asparagine residues, which can be glycosylated in the extracellular domain [16,32], were conserved. It was obvious that eqTHN had a shorter cytoplasmic tail (CT) domain than huTHN, which has also been reported in feline tetherin [33]. A previous study demonstrated that the shorter cytoplasmic tail domain in eqTHN affected neither its localization nor the lentivirus tethering function [20]. However, the fact that tetherin is localized to lipid raft domains used for budding by FLUAV suggested to us that the shorter cytoplasmic tail domain of eqTHN might affect its antiviral function. 

To investigate whether this feature contributed to the stronger restriction activity of eqTHN towards FLUAV, we constructed two MDCK cell lines, one stably expressing a huTHN-truncated mutant with the 14 N-terminal amino acids deleted (huTHN_del_14) and another stably expressing an eqTHN chimera with 14 amino acids of the cytoplasmic tail domain of huTHN added to its N terminus (hu_eqTHN). These two mutant proteins were also expressed as the wild types (Figure 2B,D). SC09 was then used to infect the control and the cells expressing tetherin and its mutants at MOIs of 0.01 or 0.1, respectively. We found that the release of SC09 was markedly inhibited by eqTHN at both MOIs, while hu_eqTHN had only very limited ability to block SC09 egress (Figure 2C). As with huTHN, huTHN_del_14 was unable to inhibit the budding of SC09 (Figure 2D). These results suggested that the higher restriction levels of eqTHN could be partly ascribed to its shorter cytoplasmic tail domain. 

Laser confocal microscopy showed both of the mutant proteins still localized on the surfaces of host cells (Figure 2E). The replacement of eqTHN CT with huTHN CT did not alter the localization of tetherin, demonstrating that the shorter CT of eqTHN was instead important in the maintenance of its antiviral activity. 

### 3.3. GPI Was Indispensable for the Tethering Function of EqTHN

**Figure 3:**
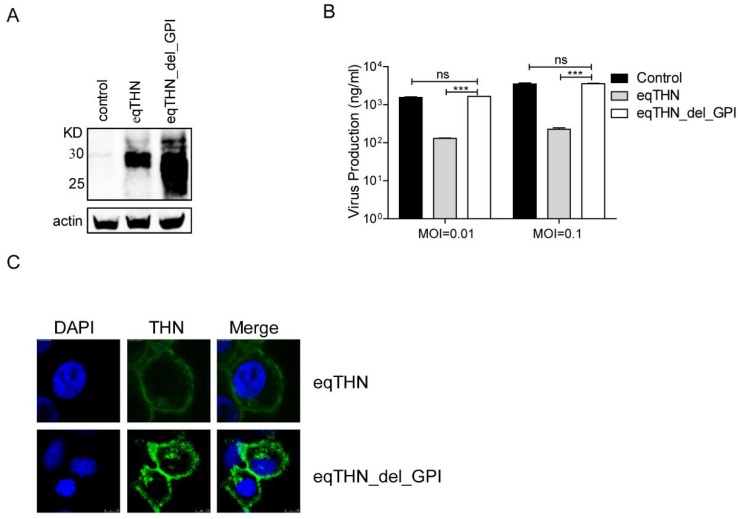
The GPI anchor was the key domain for the tethering function of eqTHN. (**A**) Stable expression of HA-tagged eqTHN_del_GPI in MDCK cells. Cells were lysed and the sample was treated as described for panel A in Figure 1; (**B**) The amount of SC09 released from MDCK and eqTHN_del_GPI-expressing MDCK cells at 48 h post-infection with SC09 at MOIs of 0.01 and 0.1. Infection and detection were carried out as described as above. The results of a single experiment performed with duplicate samples are shown, and results were subsequently confirmed in three separate experiments. Error bars indicate standard deviations (SD); (**C**) Subcellular localization of eqTHN and eqTHN_del_GPI on the cell surface with scale bars indicating 10 μm. Detection was carried out as described for panel F in Figure 2.

It is well-known that the GPI domain of tetherin is essential in the inhibition of the budding of certain enveloped viruses [16]. Previous studies have reported that the GPI anchor is crucial for the inhibition of release of FLUAV virus-like particles (VLPs) and FLUAV produced by a reverse genetics system [34], while whether the GPI is important in eqTHN for the inhibition of wild-type FLUAV is currently unclear. We examined whether the GPI domain functioned in the inhibition of wild-type SC09 release, by generating eqTHN GPI-deleted MDCK cell lines (eqTHN_del_GPI). The expression levels of eqTHN_del_GPI were higher than those of eqTHN (Figure 3A), although quantification of the number of SC09 viruses released into the supernatants showed no significant differences between the eqTHN_del_GPI-expressing and the control cells (Figure 3B). This clearly suggested that GPI was required for eqTHN activity, and, furthermore, that the function of GPI was most likely to be due to its tethering potential rather than its localization, as the eqTHN_del_GPI could be expressed on the cell surface at a higher level, shown by subcellular distribution using fluorescence microscopy (Figure 3C). Taken together, these results further confirmed that eqTHN could inhibit the release of FLUAV from MDCK cells, and that the GPI anchor was required for the tethering of nascent virus particles onto the cell surface.

### 3.4. NA and HA of HBG Could Counteract both HuTHN and EqTHN 

**Figure 4:**
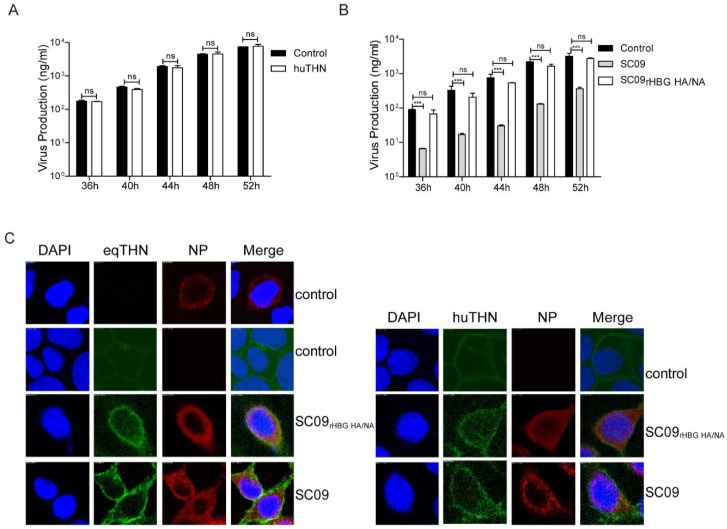
NA and HA of A/Hamburg/4/2009 (H1N1) (HBG) could counteract both huTHN and eqTHN. (**A**) The amount of SC09_rHBG HA/NA_ released from MDCK and huTHN-expressing MDCK cells at 36, 40, 44, 48 and 52 h post-infection with SC09_rHBG HA/NA_ at an MOI of 0.1. Infection and detection were carried out as described as above. The results of a single experiment performed with duplicate samples are shown, and results were subsequently confirmed in three separate experiments. Error bars indicate standard deviations (SD); (**B**) The amounts of SC09 and SC09_rHBG HA/NA_ released from MDCK and eqTHN-expressing MDCK cells at 36, 40, 44, 48 and 52 h post-infection at an MOI of 0.1. Infection and detection were carried out as described as above. The results of a single experiment performed with duplicate samples are shown, and results were subsequently confirmed in three separate experiments. Error bars indicate standard deviations (SD); (**C**) Subcellular localization of tetherins after infection with scale bars indicating 10 μm. MDCK-, huTHN- and eqTHN-expressing MDCK cells were infected with SC09 or SC09_rHBG HA/NA_, 48 h post-infection, cells were stained with an anti-HA antibody (green) and anti-nucleoprotein (NP) antibody (red), and nuclei were stained with DAPI (blue). The cells were subsequently analyzed using confocal microscopy.

It has been claimed that HA and NA of pandemic FLUAV A/Hamburg/4/2009 (H1N1) (abbreviated HBG) confers huTHN resistance [28]. We next investigated whether HA and NA of HBG counteracted eqTHN. We created an SC09 reassortant (termed SC09_r×HBG HA/NA_) equipped with the HA and NA segments from HBG. In our experiments, huTHN was unable to block the release of SC09_r×HBG HA/NA_ at an MOI of 0.1 at any of the time points assessed (Figure 4A). By contrast, eqTHN fully lost the ability to inhibit the egress of SC09_r×HBG HA/NA_, with an equal amount of virus being observed in eqTHN-expressing and the control cells (Figure 4B). The results showed HA and NA of both SC09 and HBG were able to counteract huTHN, but only HA and NA of HBG were able to antagonize eqTHN. This result illustrated that, unlike a cross-species countermeasure in different lentiviruses, human FLUAV could overcome restriction imposed by eqTHN through the surface glycoproteins HA and NA.

In order to investigate whether the infection of SC09_r×HBG HA/NA_ and SC09 caused different subcellular localization of tetherins, and whether SC09_r×HBG HA/NA_ could subsequently release effectively from tetherin-expressing cells, we studied the localization of eqTHN and huTHN in cells infected with either SC09_r×HBG HA/NA_ or SC09. There were no differences in localization between them (Figure 4C). EqTHN and huTHN still localized on the surface of cells with either SC09 or SC09_r×HBG HA/NA_ infection or without infection, which indicated that HA and NA did not interfere with their localization. However, the expression levels of eqTHN and huTHN seemed to increase after infection which could be explained by rising interferon (IFN) levels after viral infection in order to achieve the antiviral goal of the host, and eqTHN increased most with SC09 infection. We therefore speculated that the strong restriction activity of eqTHN against SC09 might be due to the fact that HA and NA of SC09 could not degrade eqTHN while SC09_r×HBG HA/NA_ could. 

### 3.5. 13T and 49L of HA, and 32T and 80V of NA Helped SC09 to Counteract EqTHN

**Figure 5:**
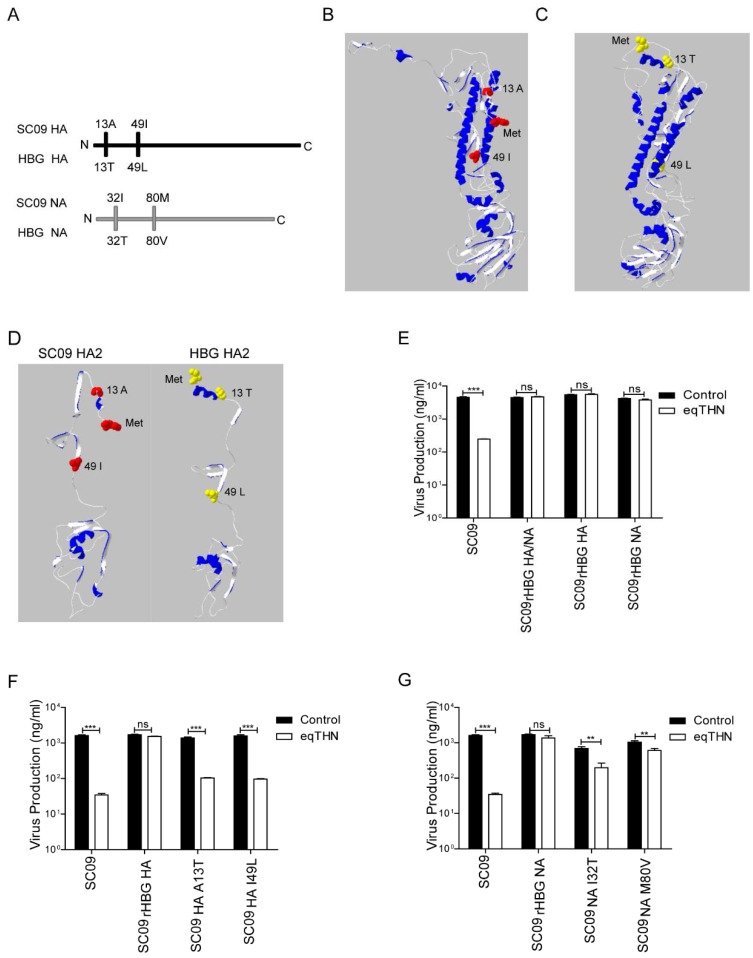
The ability of 13T and 49L in HA, 32T and 80V of NA to facilitate effective release of SC09 from eqTHN-expressing cells. (**A**) Amino acid sequence alignment of NA and HA between SC09 and HBG. Distinct sites were displayed; (**B**) Structure prediction of HA of SC09 using Phyre2. The analysis was performed using Swiss-PBD Viewer software; (**C**) Structure prediction of HA of HBG using Phyre2. The analysis was performed using Swiss-PBD Viewer software; (**D**) Structure prediction of HA2 domain of SC09 and HBG. The analysis was performed using Swiss-PBD Viewer software; (**E**) The amounts of SC09, SC09_rHBG HA/NA_, SC09_rHBG HA_ and SC09_rHBG NA_ released from MDCK and eqTHN-expressing MDCK cells at 48 h post-infection at an MOI of 0.1. Infection and detection were carried out as described as above. The results of a single experiment performed with triplicate samples are shown, and results were subsequently confirmed in three separate experiments. Error bars indicate standard deviations (SD); (**F**) The amounts of SC09, SC09_rHBG HA_, SC09_HA A13T_ and SC09_HA I49L_ released from MDCK and eqTHN-expressing MDCK cells at 48 h post-infection at an MOI of 0.1. Infection and detection were carried out as described as above. The results of a single experiment performed with triplicate samples are shown, and results were subsequently confirmed in three separate experiments. Error bars indicate standard deviations (SD); (**G**) The amounts of SC09, SC09_rHBG NA_, SC09_NA I32T_ and SC09_NA M80V_ released from MDCK and eqTHN-expressing MDCK cells at 48 h post infection at an MOI of 0.1. Infection and detection were carried out as described as above. The results of a single experiment performed with triplicate samples are shown, and results were subsequently confirmed in three separate experiments. Error bars indicate standard deviations (SD).

SC09 and HBG were both isolated in 2009 and found to belong to the same subtype. We investigated the reasons for their considerably different abilities to antagonize eqTHN. An alignment of amino acid sequences of HA and NA in SC09 with those of HBG revealed differences at only 2 positions in each protein, namely positions 13 and 49 of HA, and positions 32 and 80 of NA (Figure 5A), indicating that these sites were likely to contribute to the eqTHN resistance phenotype. Since the relationship between the protein structure and changes in protein interactions could allow viruses to replicate effectively in the host, we speculated that these sites might affect the structures of HA and NA. To verify this, we predicted the structures of HA and NA of both virus strains using the online service Phyre2 (Figure 5B,C). The differences in N-terminal direction, and the number of α-helices and β-sheets in the stalk-like HA2 domain could be seen between SC09 and HBG (Figure 5D). HA2 is known to first anchor HA to the viral membrane and then to facilitate membrane fusion of the virus with the endosomal bilayer, both of which are important for viral replication in host cells [35]. Modification of HA2 might alter its interaction with eqTHN and thus keep or even enhance its role in viral replication and release. As the structure of the N-terminal of NA has not been resolved to date, we were unable to determine whether there was a structural change in NA.

In order to analyze the effect of HA and NA individually on eqTHN resistance, we constructed an SC09-derived 7 + 1 reassortant virus expressing the either HA or NA of HBG (SC09_rHBG HA_ or SC09_rHBG NA_), to infect both eqTHN-expressing and control cells. Our results showed that release of SC09 reassortants bearing HA or NA of HBG was not modulated by eqTHN, indicating that either HA or NA alone of HBG had the ability to counteract eqTHN in an SC09 context (Figure 5E). This was consistent with a previous study regarding herpes simplex virus 2 (HSV-2), showing that tetherin could be antagonized by viral multiple glycoproteins [36]. 

Finally, we tested the contribution of each mutation on HA and NA to SC09 release under eqTHN expression. The mutant viruses were generated based on SC09 with a single point mutation A13T or I49L on HA (SC09_HA A13T_ or SC09_HA I49L_), I32T or M80V on NA (SC09_NA I32T_ or SC09_NA M80V_), and then were used to infect both eqTHN-expressing and control cells. The release of SC09_HA A13T_ and SC09_HA I49L_ was markedly inhibited compared with that of SC09_rHBG HA_, showing that viruses with a single point mutation in HA almost completely lost the ability to overcome eqTHN restriction (Figure 5F), while mutants with a single point mutation in NA could still greatly enhance release of SC09 from eqTHN-expressing cells (Figure 5G). Because the release of SC09_NA I32T_ or SC09_NA M80V_ only displayed 1.5–3 fold reduction (*p* < 0.01), compared with an around 20-fold decrease (*p* < 0.001) of SC09, SC09_HA A13T_ or SC09_HA I49L_ in eqTHN-expressing cells in comparison with the control cells. Thus, the coexistence of 13T and 49L on HA was a precondition to fully antagonize eqTHN. By contrast, 32T or 80V alone could help NA of SC09 work effectively. 

## 4. Discussion

HuTHN has broad activity against a wide range of viruses, including retroviruses [37], herpesviruses [12,38], rhabdoviruses [39], filoviruses [11], and arenaviruses [40]. To achieve replication in their hosts successfully, these viruses develop their encoded countermeasures [22,41,42]. There is strong evidence that huTHN is able to inhibit the release of FLUAV VLPs [26,34,43]. For wild-type FLUAV, however, there is only a single study showing inhibition of several seasonal and laboratory-adapted FLUAV strains by huTHN, while several pandemic viruses counteract tetherin via their HA and NA [28]. To date, the only tetherin that has been investigated with respect to human FLUAV strains has been huTHN. Here, we demonstrate that eqTHN can inhibit release of SC09 while huTHN has no effect on it, showing that there is an interspecies restriction between FLUAV and its hosts imposed by tetherin. The fact that the human FLUAV HBG can not be inhibited by eqTHN and that the equine FLUAV XJ07 is not sensitive to huTHN indicates the complex coevolution between FLUAV and tetherins.

Significant controversy exists surrounding whether tetherin is able to exert an antiviral effect on wild-type FLUAV [26,34,44]. These observed differences may be associated with cellular systems, the efficiency of tetherin expression, or even the amount of tosyl-phenylalanine chloromethyl-ketone (TPCK)-treated trypsin added to virus growth medium, which has been demonstrated to have an inhibitory role of tetherin on HIV release [45]. Furthermore, the techniques, including plaque formation assay and FFU assay to detect whether virus titers are similar in control and tetherin-expressing cells, can potentially cover up small differences after viral reinfection and replication in MDCK cells and cost more time [28,34]. Moreover, defective viruses in the supernatant that are unable to infect MDCK cells are ignored. 

The antigen capture enzyme-linked immunosorbent assay (AC-ELISA) method developed in our lab exactly overcomes the above drawbacks, enabling us to detect viruses directly, including defective viruses, in the supernatant without further infection and viral protein expression in the cells, and is more sensitive than western blot. We sampled at different time points (from 24 h to 60 h post-infection) to display the growth kinetics of the viruses in tetherin-expressing MDCK cells. The results from AC-ELISA are consistent with those of the FFU assay, suggesting that the ELISA can be used as an indirect measure of viral titers. Since the tetherin functions at the viral budding stage by tethering the virions onto the cell membrane, we would normally only be able to observe the effect from 24 h to 60 h post-infection. And with increasing viral production latterly, the tetherin was saturated and the difference of viral productions between wild-type and tetherin positive cells dwindled (Figure 1B,C).

MDCK cells are easy to infect with FLUAV, express low levels of canine tetherin and cannot even inhibit the release of VLPs of FLUAV [26], making them an ideal cell line in which to study the function of tetherin. MDCK cells expressing either eqTHN or huTHN were developed and infected with different FLUAV strains. Strong inhibition of FLUAV was observed in those cells expressing eqTHN but not huTHN. 

In contrast to retroviruses, FLUAV has the ability to cross species barriers and can transmit from one host to another. To overcome the species barrier, the virus requires several adaptations to the new host [46,47]. Theoretically, adaptive mechanisms can be divided into two categories. The first category comprises adaptations to the host, including changes in the viral HA, NA and polymerase basic protein 2 (PB2) during the adaptation of avian FLUAV to humans [48,49]. The second comprises adaptations to antagonize host restriction factors that inhibit viral replication. Our results suggest that eqTHN has a strong inhibitory activity towards both human FLUAV SC09 and equine FLUAV XJ07, while huTHN can not affect either of them. This supports a previous study showing that human, avian, swine, and equine FLUAV strains are broadly insensitive to huTHN [27]. It may be that a human can be infected by a wide range of FLUAV, whereas limited strains are able to spread between horses and the H3N8 subtype viruses are the only viruses currently circulating in horses [50].

Restriction factors are components of the intrinsic and innate host defense process and may lead to strong selective pressure against newly-invading viruses. Unfortunately, there is little information about adaptive mutations that overcome such host restriction factors and facilitate interspecies transmission of FLUAV. It is extremely difficult to find key sites of antagonism against tetherin in HA and NA, due to their high mutation rates. In the present study, we find that 13T and 49L in HA, and 32T and 80V in NA play an important role in overcoming the inhibition of eqTHN in an SC09 context. These four amino acids exist naturally in the human H1N1 strain of swine origin, HBG. This strain was isolated from a patient in Hamburg during the 2009 pandemic and then was adapted to the mouse in the lab by serial passages [51]. Adaptive mutations in the nucleoprotein of HBG to avoid restriction by human myxovirus resistance proteins Ⅰ (Mx1 or MxA) have been identified [52]. Different amino acid positions are identified in A/Brevig Mission/1/1918 and HBG NP, demonstrating the strong positive selection pressure exerted on the viruses by human MxA. The emergence of 13T and 49L in HA and 32T and 80V in NA to counteract eqTHN in our study may have also resulted from selective pressure. Of note was that HBG was only found to spread between humans. The resistance to eqTHN might be linked to a complex evolutionary history between FLUAV and its hosts, which was consistent with the ability of Ebola virus glycoprotein to counteract human, primate, and murine tetherins [53]. 

Many studies have shown that the unusual molecular topology of tetherin is important for its tethering function [10,54]. Our study shows that eqTHN both carries a shorter N-terminal region and displays strong inhibition on FLUAV release. The shorter cytoplasmic domain of eqTHN compared with huTHN leads to the loss of a dual-tyrosine motif (Y6–Y8). This region has been found to be key for the internalization of tetherin from lipid rafts on the plasma membrane and intracellular compartments by clathrin-mediated endocytosis [55,56]. Moreover, it has also been shown to be required for the introduction of the NF-κB signaling pathway in HEK293T cells, while it is dispensable for its antiviral activity [57]. However, MDCK cells are extremely insensitive to transfection reagents and it is thus difficult to transfect them. We were unable to perform this experiment to confirm whether eqTHN better activates NF-ΚB signaling pathways in MDCK cells, leading to greater antiviral activity. Murine tetherin also has a short N-terminal region, in which the first ATG is mutated, causing a higher level of expression and thus more potent inhibition of viral release [58]. Furthermore, it has been demonstrated that a shorter form of ovine tetherin possesses stronger antiviral activity than does the longer form [59]. A crystal structure of the protein complex including the viral membrane protein Vpu in HIV-1, the huTHN cytoplasmic domain and the core of clathrin adaptor protein complex 1 (AP1) lays the foundation for our understanding of how Vpu hijacks the AP1-dependent membrane trafficking pathways so that they mistraffick tetherin and cause its degradation [60], thus promoting release of the virus. However, it is unclear how a shorter N-terminal region on tetherin affects the antiviral activity and this mechanism of action needs to be further explored.

In conclusion, our current research evaluates anti-FLUAV activities of huTHN and eqTHN and finds key sites on HA and NA in an SC09 virion context to antagonize eqTHN, which gives us a better understanding of the sequence-specific interaction between FLUAV and tetherin. More work needs to be done to illustrate the detailed molecular mechanism involved in the structure-specific interaction and evolution, which may provide new targets for antiviral intervention.
